# Merkel Cell Polyomavirus Co-Infection in HIV/AIDS Individuals: Clinical Diagnosis, Consequences and Treatments

**DOI:** 10.3390/pathogens14020134

**Published:** 2025-02-02

**Authors:** Xianfeng Zhou, Chenxue Yin, Ziqi Lin, Zhangren Yan, Jiangang Wang

**Affiliations:** 1 The Jiangxi Province Key Laboratory for Diagnosis, Treatment and Rehabilitation of Cancer in Chinese Medicine, Cancer Research Center, Jiangxi University of Chinese Medicine, Nanchang 330004, China; nccdczxf@126.com (X.Z.); linziqi@jxutcm.edu.cn (Z.L.); 2 Public Health Education and Health Service Center, Jiangxi University of Chinese Medicine, Nanchang 330004, China; 3 Graduate School, Jiangxi University of Chinese Medicine, Nanchang 330004, China; 20152035@jxutcm.edu.cn; 4 Jiangxi University of Chinese Medicine Affiliated Hospital, Nanchang 330006, China

**Keywords:** Merkel cell polyomavirus, HIV/AIDS, activation, Merkel cell carcinoma

## Abstract

Merkel cell polyomavirus (MCV) was named for its role as the causative agent of Merkel cell carcinoma (MCC), which is MCV positive in approximately 80% of cases. MCV is classified as a Group 2A carcinogen, which promotes carcinogenesis by integrating T-antigen into the cell genome. The prevalence of anti-MCV antibodies in the general population can be as high as 90%. MCV typically promotes cancer by integrating T-antigen genes into the host cell genome, and 80% of MCC cases are attributed to MCV activation. In immunocompetent individuals, MCV usually remains latent after infection. However, the incidence of MCC increases significantly in immunocompromised or immunodeficient patients, such as those who have undergone organ transplantation, have chronic lymphocytic leukemia, or are living with human immunodeficiency virus (HIV) infection. Acquired immunodeficiency is a particular feature of people living with HIV. Currently, research on HIV/AIDS patients with MCV infection, clinical outcomes, and treatments is quite limited. This paper reviews previous research and systematically examines the relationship between HIV/AIDS and MCV-associated diseases, with the aim of providing valuable information for the prevention, diagnosis, and treatment of MCV in vulnerable populations.

## 1. Introduction

In 2008, Feng et al. discovered a new human polyomavirus from malignant cutaneous neuroendocrine carcinoma—Merkel cell carcinoma (MCC)—tissue through genome sequencing. The virus was named Merkel cell polyomavirus (MCV) due to its close association with MCC tumor cases. They found that in over 80% of MCC cases, MCV gene clones were integrated into MCC tumor cells, suggesting that MCV is the main culprit causing MCC [1]. In fact, ~20% of MCC cases were associated with ultraviolet (UV) radiation [2]. Whether caused by MCV or UV, MCC mainly affects the elderly and immunosuppressed individuals. Prior to the discovery of MCV, human polyomavirus had already received widespread attention. The first two types of human polyomavirus discovered in the 1970s were named after the patient’s initials, namely BK polyomavirus (BKV) and JC polyomavirus (JCV). To date, there are around 14 polyomaviruses with human hosts and relationships with pathological conditions have been found not only MCV-associated MCC but also trichodysplasia spinulosa polyomavirus (TSV)-associated TS, BKV-associated nephropathy, JCV-associated leukoencephalopathy, etc. [3]. The discovery of MCV in 2008 not only updated the limited list of human polyomaviruses but also, for the first time, established a close association between polyomaviruses and cancer development. MCC is a rare skin cancer with neuroendocrine differentiation. In addition to MCV infection, other risk factors include sun exposure, aging, and immune suppression (such as transplant recipients, lymphoproliferative tumor patients, or HIV/AIDS) [4,5]. In clinical practice, MCC manifests as skin or subcutaneous plaques or nodules, its clinical appearance is nonspecific, similar both to benign and malign skin lesions. Therefore, a definitive diagnosis of the tumor is rarely made in clinical practice [2].

The high similarity in genome structure between human polyomavirus (MCV) and human papillomavirus (HPV) suggests that MCV may integrate into host cell DNA in a carcinogenic pattern similar to HPV. In subsequent research reports, a research team found that more than 80% of MCC are MCV positive and MCV can promote clonal expansion of cancer cells by integrating viral DNA into the tumor genome, proving that MCV infection may occur earlier than MCC [6,7,8]. The study also found that virus-induced tumor formation relies on MCV large T antigen mediation, and MCV-positive MCC cell lines rely on viral tumor expression for cell diffusion and survival [9]. Evidence shows that the activation of MCV is correlated with the host’s immune suppression level, suggesting that HIV/AIDS patients, organ transplant patients, and other immunocompromised populations have a higher risk of MCV activation and cancer [10,11,12]. This review focuses on the population of HIV/AIDS patients and elaborates and discusses, from the aspects of epidemiology, MCV activation, MCC clinical characteristics, and treatment measures, providing important ideas and references for HIV co-infection, tumor development, and antiviral treatment plans.

## 2. Genomic Characteristics and Regulatory Mechanisms of MCV

**Figure 1:**
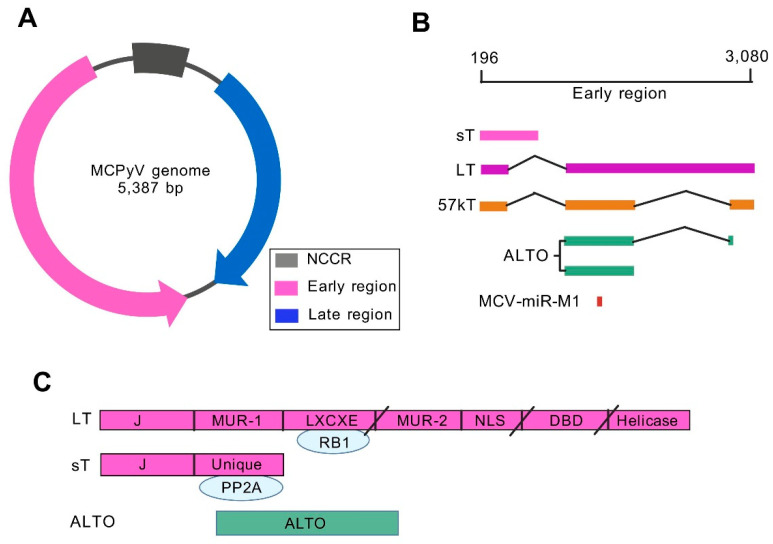
The double-stranded DNA of MCV is divided into three functional regions: early region (EVGR), late region (LVGR), and intermediate non-coding control region (NCCR), which contains bidirectional promoters and virus replication origin (**A**); the EVGR (196ȁ3080 nt) includes early region genes of LT, ST, 57 kT, and MCV-miR-M1 (**B**); Linear diagram of MCV genome for LT, sT, and ALTO (**C**). PP2A, protein phosphatase 2A; RB, retinoblastoma.

Polyomavirus is a small, non-enveloped, double-stranded DNA virus with a genome of approximately 5.4 kb, encoding 5–8 viral proteins [13]. MCV is the fifth human polyomavirus discovered and is currently one of the 14 types of polyomaviruses found to infect humans [14,15]. The genome structure of MCV is similar to that of BKV and JCV (Figure 1A), with its circular genome divided into three functional regions: early region (ER), late region (LR), and intermediate non-coding control region (NCCR) [16]. The early region encodes alternating frames of large T antigen (LT), small T antigen (sT), 57 kT antigen (57 kT), the alternative large T open reading frame (ALTO), and miRNA MCV-miR-M1. LT and ST share one N-terminal DNAJ or J domain. LT also contains LXCXE or RB binding motifs, MCV unique regions (MUR) −1 and −2, nuclear localization signal (NLS), DNA binding domain (DBD), and helicase domain. In MCC, mutations in LT result in truncation after LXCXE, NLS, or Ori binding domain (OBD), indicated by slashes in Figure 1C. Late regions are transcribed in opposite directions and encode viral capsid proteins VP1 and VP2 [16]. NCCR contains replication origin and bidirectional promoter elements that control the transcription and expression of EVGR and LVGR [12]. The two main splicing products, LT and sT antigens, are involved in the viral infection cycle, cell cycle progression, and malignant transformation of host cells [9,17]. MCV-miR-M1, which targets the oncogenic MCV LT, has previously been shown to play an important role in the establishment of persistent infection [18]. The MCV T antigen gene is considered a viral oncogene involved in cell transformation and has received much attention [19]. The continuous expression of C-terminal truncation of MCV oncogene sT and LT is essential for the development of MCC [20]. Hesbacher et al. found that inactivation of RB1 is the predominant function of MCV LT that promotes cell growth during MCPyV-driven tumorigenesis [21]. In addition, sT has the potential to promote cell transformation through enhancing LT-t protein levels in MCC cells [22]. Transcriptomic and proteomic analyses indicated that the sT-MYCL-EP400 complex may contribute to cell transformation and MCPyV oncogenic potential [23]. Although these findings demonstrate that MCV T antigen is the main pathogenic factor of virus-positive MCC, it is unclear whether NCCR will affect infection outcomes and participate in carcinogenesis. 

Previous studies have shown that a significant feature of NCCR is the occurrence of rearrangements [24,25]. NCCR rearrangement is believed to originate from sustained activation in the host and is typically associated with disease manifestations [3]. For example, polyomavirus-associated nephropathy (PVAN) related to BKV reactivation or progressive multifocal leukoencephalopathy (PML) caused by JCV is highly associated with NCCR rearrangement [26,27]. When virus replication occurs without an effective host cell immune response, NCCR mutations or rearrangements can also occur in vitro [28]. Therefore, NCCR rearrangement under host immune suppression affects early and late gene transcriptional regulation, but there are currently very limited data on the association between MCV NCCR rearrangement and the development of MCV-related diseases. However, previous studies indicated that BKV reactivation was associated with sequence variability within transcription factor binding sites (TFBS) in the rearranged NCCR [29]. Prezioso et al. identified several putative TFBS in MCV and deletions, insertions, or single base substitutions altered the NCCR canonical configuration in MCV strains from HIV/AIDS patients with unclear impact on their pathogenic features [12]. Hashida et al. evaluated the genetic variability of MCV NCCR in skin swabs from healthy individuals of different races and regions in Japan, indicating that insertions and deletions can be used to classify NCCR into five genotypes. According to this classification, tandem repeats were only found in the NCCR of Japanese MCC patients, while white individuals from Europe or North America exhibited other genotypes [20]. In vitro experiments have shown that the TFBS Sp1 in NCCR is a key factor in the bidirectional expression regulation of BKV virus ER and LR [30]. Therefore, future research on TFBS-regulated gene expression in MCV NCCR is worth paying attention to. However, potential regulation mechanisms of BKV latency/reactivation by viral gene NCCR (viral reactivation), sT (viral gene downregulation), miRNA (immune evasion), and Ango (impair innate immunity) were clarified [3]. 

## 3. Epidemiological Characteristics of MCV

MCV is currently the only human polyomavirus directly associated with tumorigenesis. In over 90% of MCC cases, monoclonal integration of viral DNA into the tumor cell genome leads to clonal amplification and carcinogenesis [31]. The anti-MCV serum antibody IgG of the general healthy population is 46–88%, and it increases with age, indicating that people are easily exposed to MCV [32,33]. 75%–80% of MCC tumors contain MCV virus particles. In addition to MCC tumor tissues, MCV has also been detected in non-MCC patients’ skin, oral cavity, liver, lungs, kidneys, and other tissues, indicating that MCV is widely distributed in the human body [34]. In recent years, a high prevalence of MCV has also been found in respiratory and fecal samples of immunocompromised patients, increasing important information on the prevalence, persistence, and transmission routes of MCV in the respiratory and gastrointestinal tracts [4,6]. There are studies indicating that MCV may be involved in the development of non-small cell lung cancer (NSCLC), and immunohistochemical analysis has found that MCV large T antigen is expressed in NSCLC tumor cells [35].

The results of serological antibody surveys conducted in various countries around the world show that early exposure to MCV infection is likely to occur in childhood, due to the low seropositivity rate in children and the significantly increased seropositivity rate in adults. A study in Italy showed that the MCV serum positivity rate significantly increases with age in the general population aged 1–100, ranging from 41.7% in 1–4-year-olds to 87.6% in 15–19-year-olds, and remains at 79.0–96.2% in adults [32]. We tested the levels of BKV, JCV, and MCV antibodies in 810 HIV/AIDS individuals and 810 healthy controls and found that the positive rate of MCV antibodies also increased significantly with age, with an overall positive rate of 65% [36]. Healthy individuals typically do not produce antibodies against MCV LT and sT antigens, while they can be detected in MCV-positive MCC patients [37]. Another important point is that the titer of T antigen antibodies significantly decreases after effective MCC treatment, which can serve as a biomarker for disease status. It is worth noting that MCC patients have relatively higher titers of anti-VP1 antibodies compared to the general population [38].

## 4. HIV Infection Increases the Risk of MCV Activation and MCC Occurrence

Merkel cells are found in the epidermis (outer layer of the skin). They were originally described in the late 1800s by the German anatomist Friedrich Merkel. The cells are present in human skin at varying levels according to body site, they are at highest density on the fingertips and lips/face where touch sensation is most acute [39]. Merkel cells are a unique type of cell that exhibit features of both epithelial and neuroendocrine cells, with important epithelial markers including AE1/AE3, CAM 5.2, pan-cytokeratin, and Ber-EP4 [40]. These epithelial markers play a crucial role in identifying and characterizing MCC and other neuroendocrine neoplasms. Given that MCV is very common while MCC is very rare, it is likely that other risk factors play a role in the development of this cancer. In addition to UV, older age and a weakened immune system, MCC usually arises in people who have light-colored skin [2]. 

Although most people have been naturally infected with the MCV virus, the likelihood of developing MCC due to this infection is very low in populations with normal immune function [36]. However, factors such as immune suppression can reactivate the virus and promote viral gene integration, mutations, and cellular carcinogenesis [41,42,43]. The occurrence of MCC tumors is quite similar to that of Kaposi’s sarcoma (KS) and Burkitt’s lymphoma (BL), both of which are more prevalent in populations with immune dysfunction [44]. Overall, patients with immune dysfunction often experience rapid onset and progression of these tumors, which can ultimately lead to more severe health conditions [45]. Immune dysfunction is a significant factor in the development of MCC tumors. It can increase the risk of developing MCC, and once a patient progresses to this stage, their prognosis tends to be worse [46,47]. Patients with systemic immune dysfunction often experience a poor quality of life, with a three-year survival rate that is over 50% lower compared to immunocompetent patients with MCC [48].

**Figure 2:**
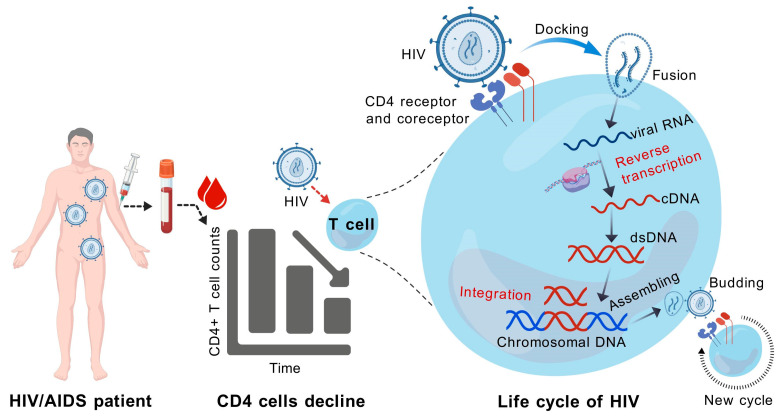
Diagram of HIV infection that causes CD4+ T cells decline and HIV life cycle in CD4+ T cell.

A study conducted in the United States found that the risk of developing Merkel cell carcinoma (MCC) in individuals with HIV/AIDS is 13.4 times greater than that in the general population. This suggests that immunodeficiency may facilitate the activation of Merkel cell polyomavirus (MCV) and contribute to the onset of MCC [49]. HIV infection begins when HIV encounters a CD4 cell. The seven steps in the HIV life cycle are: (1) binding to CD4 receptor and coreceptor; (2) fusion; (3) reverse transcription; (4) integration; (5) replication; (6) assembly; and (7) budding (Figure 2). Untreated HIV replication causes progressive CD4 + T cell loss and a wide range of immunological abnormalities, leading to an increased risk of infectious and oncological complications [50]. 

To date, there have been no direct reports on the relationship between CD4+ T cell counts and pathogen-specific survival of MCC in individuals infected with HIV. However, it has been established that patients with immune dysfunction are at a higher risk for developing MCC due to MCV activation [51]. Recent studies indicate that HIV-infected individuals with severe immune dysfunction exhibit significantly higher levels of MCV DNA in their skin compared to those with mild immune dysfunction [52]. Furthermore, another study revealed that the MCV DNA load in the serum of HIV-1-positive patients not receiving antiviral treatment was significantly higher than that of HIV-1-negative individuals, with a p-value of less than 0.01 [53]. Passerini et al. detected both human papillomavirus (HPV) and human polyomavirus in anal swab samples from 150 HIV-positive individuals. They discovered that the proportion of patients with both HPV and MCV was the highest and that the viral load of MCV was significantly elevated among HIV-positive individuals with HPV, with a p-value of less than 0.0001 [54]. Additionally, MCC tumors tend to progress rapidly and adversely affect the quality of life in HIV/AIDS patients. Notably, the onset of MCC occurs nearly 20 years earlier in HIV/AIDS patients compared to those with a normal immune response. An epidemiological study in the United States in 2016 suggested that the HIV-MCC population is predominantly composed of young adults, with no patients over the age of 65 [55]. In summary, patients with HIV/AIDS who are infected with MCV exhibit a higher incidence of MCC compared to healthy individuals.

## 5. MCC Occurrence in HIV/AIDS Individuals

Although MCC is a highly aggressive neuroendocrine carcinoma, early diagnosis and treatment can lead to a better prognosis. Ramachandran et al. reported a case of atypical Merkel cell polyomavirus (MCV)-positive MCC in an HIV-positive patient in the United States. Initially, a fixed subcutaneous mass measuring approximately 8 cm was discovered on the patient’s left forehead, lacking the typical features of MCC, and it was later found to have metastasized to the lungs and liver. The patient underwent palliative radiotherapy and cisplatin/etoposide chemotherapy for brain involvement, followed by immunotherapy [56]. The prevalence of anti-MCV serum antibodies in the general population ranges from 46 to 88%, with most individuals presenting asymptomatically during latent infections [31]. However, MCV can be activated in immunocompromised individuals, such as those with HIV/AIDS, which may lead to the development of diseases. Therefore, implementing more effective strategies to enhance the T cell immune responses of patients is crucial in preventing MCV activation. The combination of interleukin-2 and antiviral therapy has been recommended to hinder the metastasis and spread of MCC tumors in HIV/AIDS patients. Immunosuppressive agents, such as statins, are believed to triple the risk of MCC, indicating a significant increase in the likelihood of MCV activation under immunosuppressive conditions [57].

While there are isolated clinical cases of MCC reported in China, relevant research on MCV infection, activation, and pathogenesis in HIV/AIDS populations has been lacking. Recently, our team has embarked on conducting serological surveys for human polyomaviruses and monitoring viral load in HIV/AIDS populations. We have established methods for ELISA antibody detection and nucleic acid quantification based on virus-like particles (VLPs). Our findings indicate that serum MCV viral load is inversely proportional to CD4+ T cell count, suggesting that immune deficiency promotes MCV activation [36]. Two other immunodeficiency-related carcinogenesis KS and BL were related to human herpesvirus 8 (HHV8) and Epstein–Barr Virus (EBV), respectively [58,59]. KS has a variable clinical course ranging from minimal mucocutaneous disease to widespread organ involvement. KS regression is likely under the control of HIV with antiretrovirals [59]. However, HIV might have either a direct or indirect oncogenic role in BL progression [58]. Given these insights, it is essential to distinguish these tumors in HIV/AIDS patients by their clinical presentations and molecular/immunology diagnosis, and then specify the most appropriate clinical management. 

## 6. Clinical Characteristics of MCC

**Figure 3:**
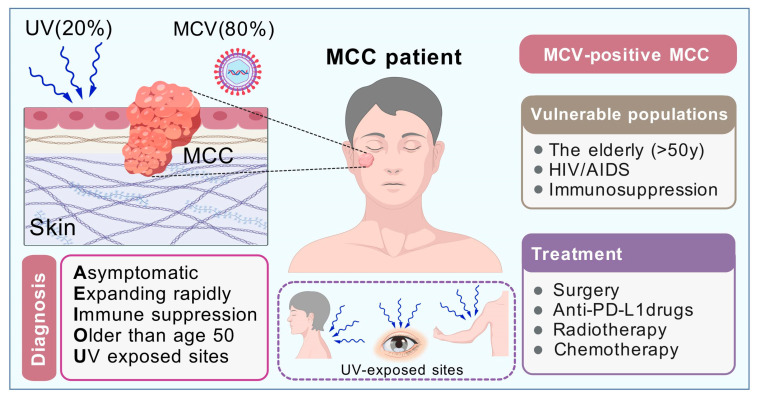
Diagram of risk factors, clinical diagnosis, vulnerable populations, and recommended treatments of MCC. Clinical diagnosis with the “AEIOU” principle and treatments recommended are listed for MCC. MCC mainly occurs in UV-exposed sites including face, limbs, hands, eyelids, etc. Given the complexity, invasiveness, and individual differences of each case, MCC is best treated by a multidisciplinary team.

Regardless of HIV infection, MCC is a highly invasive primary neuroendocrine carcinoma of the skin. Most cases (~80%) of MCC are closely linked to MCV, while the remaining cases are often triggered by excessive exposure to UV (~20%), leading to mutations in skin basal cells and carcinogenesis (Figure 3) [44]. Typically, MCC presents as glossy, flesh-colored, or bluish-red intradermal nodules, which may be accompanied by ulcers or scabs. In most instances, MCC tissues test positive for MCV nucleic acid [60]. An important study evaluated the typical manifestations of MCC, identifying characteristics such as being asymptomatic or non-tender, rapid expansion, immunosuppression, occurrence in individuals over 50 years of age, and exposure to ultraviolet radiation [47]. Despite the rapid growth of the tumors in the first three months, many MCC tumors that present as asymptomatic pink or red lesions are considered benign. Previous studies have shown that factors such as HIV-1 infection, immune suppression caused by chronic inflammation, solid organ transplantation, and hematological malignancies significantly increase the risk of MCV infection developing into MCC (Figure 3) [11,44,54]. In addition, a recent study indicated that germline variants in ATM, MAGT1, BRCA1 and BRCA1, and TP53 were associated with MCC predisposition [61]. Although the incidence rate of MCC is one-tenth that of malignant melanoma, the survival rate for MCC is considerably lower, making it the most deadly form of skin cancer. Over the past two decades, the mortality rate associated with MCC has continued to rise. In 1992, cytokeratin 20 (CK20) was identified as a key immunohistochemical marker for MCC, significantly advancing its discovery and diagnosis [60]. 

Clinicopathological features help diagnose and differentiate types of MCC. Luo et al. investigated the clinicopathological features, differential diagnosis, and prognosis of 10 MCC tissues, and found intradermal MCC of the skin displayed a nested, cord-like, cribriform distribution, polygonal cells, uniform size, and a lack of cytoplasm. Tumor cell nuclei were large and round, with clear nuclear membranes, fine and scattered chromatin, and an absence of nucleoli. They found that all cancer cells expressed CKpan, synaptophysin, and CD56, with 70% positive for CK20 [62]. Recently, Valiga et al. identified Merkel cell hyperplasia (MCH) from intraepidermal MCC by histopathologic and immunohistochemical features, in which MCH was identified by its small intraepidermal nest of monomorphic cells without atypia or mitoses, while MCC demonstrated large nests of pleomorphic cells with frequent mitoses and apoptosis [63]. Currently, recommendations for diagnostic workup, clinical stage, and treatment options for MCC patients are being updated by the National Comprehensive Cancer Network (NCCN) [39]. Epidemiological data indicate that there are approximately 2500 new cases of MCC in the United States each year, with around 1000 people succumbing to the disease. The high mortality rate is primarily due to the absence of a standardized treatment method that can enhance overall survival rates and reduce the metastasis of cancer cells [60]. Current evidence suggests that the tumor immunogenicity of MCC makes immunomodulatory therapy strategies particularly promising.

## 7. Molecular Characteristics and Clinical Treatments of MCC

Surgical treatment is considered the standard approach for local Merkel cell carcinoma (MCC). It is currently recommended to extensively remove lesions along with the sentinel lymph nodes situated 1–2 centimeters around the lesions and at deep margins. Most international guidelines advocate a tumor grading strategy that incorporates local adjuvant radiotherapy to improve cure rates [64]. Currently, there is no routine clinical diagnosis to distinguish between virus-positive (VP) MCC and virus-negative (VN) MCC, hindering the therapeutic options [65]. However, next-generation sequencing (NGS) provided a clear profile of mutational differences in key mutations in RB1, TP53, NOTCH1, and FAT1 between VP- and VN-MCC [66]. Recently, Starrett et al. clarified the global somatic alterations of a cohort of 71 MCC patients and found UV mutational signatures were detected in 24 cases characterized by highly enriched missense and truncating mutations of RB1, TP53, KMT2D, NOTCH1, NOTCH2, and FAT1 [65]. 

**Figure 4:**
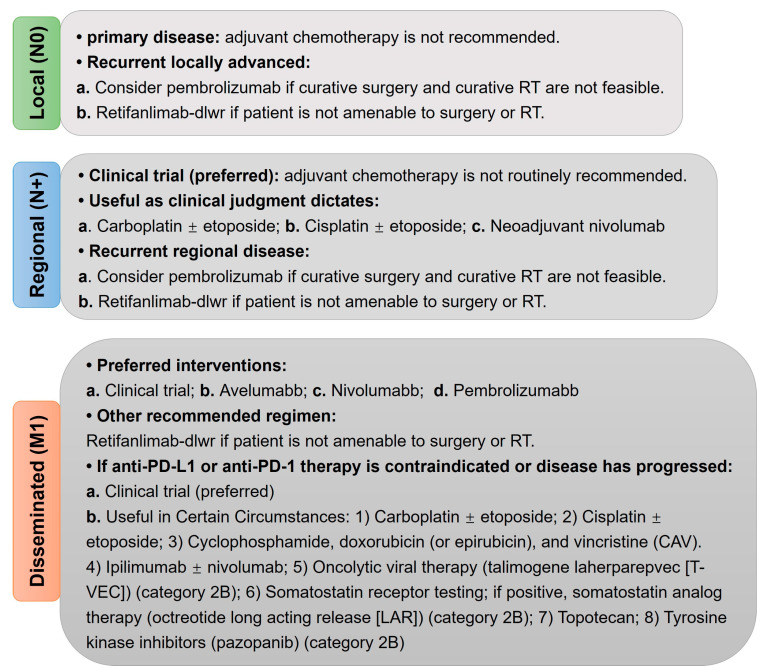
Principles of systematic therapy of MCC under the NCCN guidelines (Version 1.2023). N0: no regional lymph node metastasis detected on clinical and/or radiologic examination; N+: regional lymph nodes cannot be clinically assessed (e.g., previously removed for another reason, or because of body habitus); M1: distant metastasis detected on clinical and/or radiologic examination; RT: radiation therapy.

Research has suggested that for some newly diagnosed MCC cases, the patient’s immune suppression status—such as that seen in individuals with AIDS or chronic lymphocytic leukemia—should be considered as part of various diagnostic tests for MCC. It is also recommended to evaluate the inclusion of immune checkpoint inhibitors (ICI) for patients who exhibit symptoms of MCC (Figure 3) [47]. For late-stage, metastatic, and recurrent cases of MCC, checkpoint blockade inhibitors targeting programmed cell death protein 1 (PD-1) and programmed cell death ligand 1 (PD-L1) have shown significant activity, including sustained drug responses [67,68]. Based on these findings, these drugs have emerged as the preferred treatment option for advanced MCC. The highly responsive, virus-positive MCC cases benefiting from ICI may be related to the presence of viral tumor antigens, although conclusive evidence supporting this relationship is lacking. Despite the numerous observed differences in mutation rate and number of predicted neoantigens both VP- and VN-MCC tumors have shown high response to PD-L1 and PD1 checkpoint blockade therapy [67,68]. For MCC patients who either do not respond or cannot tolerate anti-PD-(L)1 therapy, chemotherapy remains the preferred alternative [60]. Kaufman et al. conducted a multicenter phase II clinical trial in 2016, which included participants from North America, Europe, Australia, and Asia, to assess the therapeutic effects of the anti-PD-L1 monoclonal antibody avelumab on 88 phase IV MCC patients [68]. The study found that 28 patients (31.8%) achieved objective remission, comprising 8 cases of complete remission and 20 cases of partial remission. This is currently the largest multicenter clinical study in terms of sample size, and the results indicate that avelumab offers a favorable therapeutic response and tolerability, making it a viable treatment option for advanced MCC. Additionally, the latest NCCN guidelines (Version 1.2023) recommend the principles of MCC therapy for different-stage MCC (Figure 4).

Despite the endorsement of anti-PD-L1 drugs as the recommended treatment for MCC, the effectiveness of HIV antiviral therapy means that most clinical trials tend to exclude HIV-positive patients [69]. Consequently, there are very limited data concerning the use of ICIs in this specific population. A recent small-sample clinical study in the United States found that three individuals with chronic HIV infection receiving ICI treatment for MCC demonstrated positive responses to PD-L1 drugs, suggesting these agents may be appropriate as first-line immunotherapy for MCC in HIV/AIDS patients [70]. In summary, given the complexity, invasiveness, and individual differences of each case, MCC is best treated by a multidisciplinary team [71]. Moreover, it is very important to consider the conflict or adverse immune effects of antiretroviral therapy with ICI therapy. In addition, continuous monitoring of immunological markers is recommended during ICI treatment.

Although ICI therapy has been utilized in clinical trials and treatments for MCC, a randomized controlled trial examining immunotherapy specifically for HIV/AIDS patients with MCC is currently lacking both domestically and internationally. In the absence of guided treatment data, most HIV/AIDS patients with MCC receive therapies similar to those for HIV-negative patients, including combinations of surgery, radiotherapy, chemotherapy, and immunotherapy [5]. Furthermore, most clinical trials involving immunotherapy do not include patients with HIV/AIDS, immunosuppression, or hematological malignancies [68]. However, existing treatment protocols for HIV/AIDS-related lymphomas and Hodgkin’s disease follow the same methodologies as those applied to HIV-negative patients [72,73].

## 8. Conclusions

MCV is widely prevalent, with a generally high infection rate among the population that increases with age. Activation of the virus often requires certain preconditions, including immunosuppression, HIV infection, and advanced age. Due to the low incidence of MCC and the limited diagnostic capabilities, clinical and molecular biological research on MCV activation and its role in carcinogenesis face significant challenges. AIDS is a major infectious disease closely associated with KS, BL, MCC, and other malignant tumors. There is a pressing need to promote early screening, diagnosis, and treatment of MCV activation in HIV/AIDS patients, making this a topic for further exploration in the future.

Even with treatment, MCC commonly metastasizes beyond the skin [74]. MCC tends to travel first to nearby lymph nodes. Later it may spread to your brain, bones, liver, or lungs, where it can interfere with the functioning of these organs. Therefore, differential diagnosis is vital for follow-up treatment. For instance, cutaneous mеtаѕtaseѕ of SCLC do not stain with CK20 but are positive for CΚ7, neuron-specific enolase, and thyroid transcription factor-1 [75]. As for precision treatment, it is important to identify whether it is VP-MCC or VN-MCC for subsequent treatment options. Deep sequencing can not only distinguish tumor heterogeneity but also allow the creation of optimized treatment plans based on the type of somatic mutations and the characteristics of viral gene integration.

In the long term, establishing sensitive and specific diagnostic methods is crucial for effective clinical diagnosis. This has significant public health implications for understanding MCV infection, activation, and the diagnosis and treatment of patients with weakened immune systems like HIV/AIDS patients around the world. Additionally, based on existing clinical research regarding surgical resection and immunotherapy, exploring comprehensive treatment plans for various stages of MCC—utilizing interdisciplinary techniques such as multi-omics—could greatly enhance patients’ quality of life and is a promising direction for further research.
